# Deficits of Motor Intention following Parietal Lesions

**DOI:** 10.1155/2002/310138

**Published:** 2002-08-01

**Authors:** Christopher L. Gore, P. Dennis Rodriguez, Gordon C. Baylis

**Affiliations:** University of South Carolina Department of Psychology University of South Carolina Columbia SC 29208 USA

## Abstract

Patients with lesions to the right parietal lobe were tested on their ability to reach to targets, or to respond verbally to targets. The targets occurred at the same two spatial locations -- to the left and right of the patient—with the task being cued by the color of the target. Patients were able to perform both tasks separately rapidly and without error. However, when the two tasks were interleaved, they had difficulty making a response in the left (contralesional) field when this was different to a response that they had just made. These results suggest that lesions to the parietal cortex may cause a deficit in the coding for motor intention, as well as attention in the contralesional field.

## 1. Deficits of motor intention following parietal lesions

Unilateral neglect is a syndrome associated with unilateral damage to the brain and is typically the result of an occlusion of the middle cerebral artery. This syndrome is more typically seen, and the symptoms more pronounced after damage to the right parietal lobe. Clinically, unilateral neglect may be defined as a deficit in exploration of the side of space contralateral to the lesion when other factors such as low-level sensori-motor impairments cannot account for the symptoms [9]. Having the patient engage in spatial tasks such as clock drawing, line bisection, or target detection is typically used to assess it. In a clinical setting neglect is typically seen as applying to stimuli presented to the contralesional side (left side for a patient with damage to the right hemisphere) of the patient or to actions performed in the contralesional side of space.

Many studies have investigated what representation of space is damaged in neglect (e.g. [13–15,32]). These studies have often produced conflicting results suggesting that the reference frame of neglect is related to the body, or to objects. In fact recently, Baylis and Gore [4] have shown that the reference frame of neglect can simply shift according to task instructions.

Although different studies have suggested different reference-frames for neglect, typically they focus on the spatial nature of the deficit. This is in accord with studies of responses of single cells within the parietal lobe. The parietal cortex of the macaque has been shown to contain neurons that together compute a viewer-based or a scene-based representation of space from the retina-based representation that is present in earlier parts of the visual system [1,34]. This representation of space is multimodal, including inputs from the auditory system. It is clear that such a representation of space may provide the crucial neural substrate for the allocation of attention in space. It is straightforward to see how unilateral damage to such a system could lead to inattention to the contralesional side of space as seen in neglect. Furthermore, deficits seen in reaching in neglect (e.g. [17]) can be explained purely in terms of a distortion of visuo-spatial representations.

Nonetheless, a considerable body of work in the infrahuman primate has suggested that cells within the inferior parietal lobule have a more motor-related role in reaching or other goal-directed actions (see [25]). It was initially suggested that these neurons are not involved in the generation of reaching. Similarly, patients with neglect showing motoric problems were typically explained in terms of damage to frontal areas, with neglect due to parietal lesions being characterized as purely perceptual (see, for example [10,11]). However, more recently elucidated connections between the parietal cortex and premotor areas of the frontal cortex

ISSN 0953-4180/01/02/$8.00 © 2001/2002 – IOS Press. All rights reserved mean that a role in initiating reaching actions may be possible (e.g. [18,21,30]). What remained unclear was the relation of cells that responded during a reaching action, to cells that apparently computed a viewer-based representation of visual space.

Recently, a more unified view of the parietal cortex has emerged from work by Andersen and colleagues [12,24,28,29]. This work suggested that although a viewer-based representation of space is computed in parietal cortex, many neurons code far more than a strictly perceptual representation of space. In a number of studies these workers have recorded the activity of single neurons in the parietal cortex of awake, behaving macaques. They showed that single neurons are not just sensitive to the location in space of a target item, but also sensitive to the action that must be performed towards that target (in their experiments either a saccade or a reaching action). Snyder et al. [29] interpreted these results to suggest that the parietal lobe codes spatial intention, rather than simple perceptual attention. Evidence for activation of the parietal lobe related to the initiation of a new motor program has been found in people using PET [19]. In this study participants had to change between in-phase and out-of-phase bimanual flexion. A change between programs led to parietal activation that can be considered analogous to the intention-related activity found by Andersen and colleagues. These results suggest that the parietal cortex may represent space in terms of the actions to be performed. Such a representation of space is clearly in accord with recent action-based theories of attention (e.g. [33]).

At the same time, studies of patients with damage to the parietal cortex have shown that specifically motoric problems can be associated with posterior damage. For example, Husain et al.([16]; see also [22]), required patients with parietal lesions to make responses to items at the same spatial location, by initiating a reach from different start locations – to the left and right of the target. They found that even with damage confined to the inferior parietal lobule, patients had difficulty programming leftward reaches. These results make a clear and convincing case for a role of the inferior parietal lobe in motor programming.

The present study is to test whether patients with lesions to the parietal lobe show deficits of intention in another sense. We are here interested in whether items in space are represented not just in terms of their spatial location, but in terms of the type of action that they signal (in accord with the work of Andersen and colleagues outlined above). If the parietal lobe represents space in this way, then the deficit resulting from parietal lesions would compound contralesional inattention when a new action has to be performed. In other words, not only would patients with parietal lesions be expected to show a problem paying attention to an item in the contralesional field, they would find it especially hard to carry out an action to that item if the action was new. We examined this possibility by requiring patients to carry out tasks where two different actions were possible (speaking location or reaching). We predicted that, based on the results of Snyder et al. [28, 29], these patients might have difficulty in representing the correct action to perform.

## 2. Methods

### 2.1. Participants

Five patients from HealthSouth Rehabilitation Hospital were recruited for this experiment. The lesion localization, sex, and age and brief clinical details are given below, and lesions are reconstructed from CT or MRI scans using the method of Rorden and Brett [26] in Fig. 1.

BK was a 60 year old African American female who presented with left hemiparesis, especially of the upper body, and left facial droop, but no somatosensory loss or extinction was seen. She showed no perceptual deficits in audition or taction, but severe visual extinction to clinical confrontation. A battery of tests for neglect, including line-cancellation, line bisection and clock-drawing revealed very slight evidence of neglect. She was well oriented with normal cognitive functioning, and visual fields were intact. No perceptual or attentional deficits in the auditory modality were seen in BK or any of the patients who took part in this study. A CT scan showed no acute damage, and a follow-up scan was not possible in this patient.

GL was a 75-year-old Caucasian male who had suffered two right cerebrovascular accidents (CVAs) at nine months and at one month prior to testing. Together these two infarcts produced a large lesion to the parietal and occipital lobe extending from cortex through white matter, and in places to the ventricles. At the time of testing he showed left neglect, as assessed by our battery of behavioral tests. Acutely, he demonstrated right deviation of gaze, although this had resolved at the time of testing. He was well oriented, with good memory. He showed severe left hemiparesis, including left facial paresis, and left somatosensory loss.

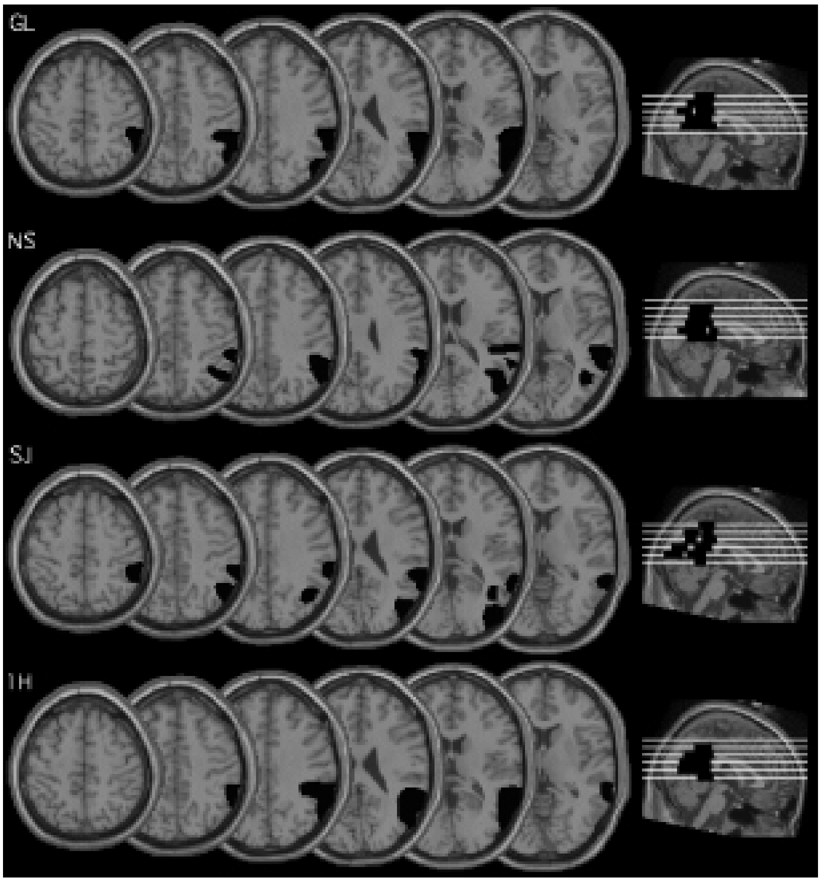

Fig. 1. The extent of the lesions for four of the patients, reconstructed according to the method of Rorden and Brett (2001) from CT (SJ, TH) or MRI (GL, NS) scans. No lesion was seen with a CT scan in the case of BK, and no later scan was possible.

NS was a 81 year old Caucasian female with an embolic lesion to the right parietal and occipital lobe. She was well oriented with some left lower-body weakness. Although she initially showed severe visual neglect as assessed by line-cancellation, line bisection and clock drawing, at the time of testing she showed no neglect. However, at the time of testing she displayed moderate extinction to clinical confrontation. She had normal acuity with small peripheral field defects on the left hemifield. All testing was carried out outside of these areas. In addition to visual extinction, she showed severe tactile extinction.

SJ was a 53 year old Caucasian male with a small ischemic lesions to the right parietal lobe. He was well oriented, and had some left-sided weakness, especially to his upper body and face. He showed some visual neglect and severe visual extinction to clinical confrontation, although visual acuity was normal. He also showed severe tactile extinction in his left upper body, along with slight tactile sensory loss on his left side.

TH was a 67 year old Caucasian male with an embolic lesion to the right parietal lobe, that had occurred three years prior to testing. He was well oriented, and showed very slight tactile extinction on his left hemibody (as far as could be tested, given that he had undergone two above-the-knee amputations), and visual extinction on clinical confrontation. No paresis was noted in this patient.

### *2.2. Apparatus and stimuli*

The apparatus is a $51 \times 51$ cm stimulus board consisting of 13 stimulus keys, shown drawn to scale in Fig. 2. In all the testing carried out here only 4 of these keys – the closest start key, a central fixation key, and one key to each side of this – were used. The keys used in this study are shown black in this figure, and those unused are shown white. All keys were translucent and could be illuminated red, green, or yellow by a bicolor LED located beneath them. The board was tilted towards the participant at a 35-degree angle. The key closest to the participant (the "start key") was illuminated with a yellow LED. Beneath each key was a microswitch that detected a slight touch on the key. The onset of LEDs and detection of closure of the microswitches was accurate to within less than 1 msec. The stimulus board was interfaced to a Dell Pentium computer that recorded target positions, response times (RTs) and errors. The responses of young and older adults using this apparatus have been well-documented [3,4,27].

### 2.3. *Design*

There were two tasks that were carried out initially in a blocked design, and later interleaved (see below). In the reach task the patient had to reach to touch the key illuminated red to the left or the right of the fixation key. In the say task, the patient had to keep their hand on the start key and say which side a green key was illuminated. Within each block of 48 trials, there were equal numbers of trials in which the key on the left, or that on the right was illuminated. For each task trials were sorted both according to whether the target was (with equal probability) on the left or right (the factor side), and in terms of whether the target key was (with equal probability) on the same or different side from the previous trial (the factor side change). These two factors, plus the third factor of which task was performed (the factor task) formed the basis of the analysis of the data. The order of conditions was determined according to a pseudorandom order that was different for each patient.

After initial testing with the two tasks individually, the patients switched to performing these tasks in an interleaved manner. Within each block of 48 trials there were equal numbers of trials in the left and right field, and equal numbers within each of these were reach and say tasks. Trials were sorted according to the three factors of side, side-change and task as before. To these was now added a fourth factor task-change, which was whether the task (reach or say) was the same as on the previous trial.

### 2.4. *Procedure*

The sequence of events within each task was explained to each patient at the beginning of that task. The patient was instructed to look at the central illuminated key on the board ("fixation key"). The experimenter monitored that the patient maintained fixation during this time, and pressed a key to abort trials if this condition was not met. This occurred on approximately 3% of trials. The patient, fixating the central key, then pressed the start key on the reach apparatus to initiate trials. After a delay of 500 msec, one of the keys on the board was illuminated as the target key. In the case of the reach task (when the target was illuminated red), the patient was instructed to reach out and touch the target key as quickly as possible. A reaction time was measured as the total time from stimulus illumination to touching the target key. In the case of the say task, patients were required to maintain pressure on the start key, and say "left" or "right" according to the location of the target. The experimenter pressed the <space> bar of the keyboard as soon as the patient spoke, then entered the response as a keypress on the "Z" key (if the patient said "left"), or the "?" key. The experimenter was seated facing the patient and could not see the reaching apparatus during trials. Thus variance in the reaction time of the experimenter might lead to a reduction in the experimental power. However, this variability was minimized by not requiring a choice by the experimenter, but simply an initial speeded response that the patient had spoken. The patients were encouraged to rest at any time, although they never chose to exercise this option, except between blocks of trials. No feedback on performance was given during the testing, apart from general encouragement.

After performing the two tasks individually, each patient then went on to perform the two tasks in a randomly interleaved manner.

### 2.5. *Results – blocked tasks*

All five patients carried out these individual tasks with no errors, and so analyses were all carried out on the RTs of response. The RTs of the five patients in these two tasks are shown in Fig. 3. An analysis of variance (ANOVA) of the patient median RTs showed an effect of the side on which the target appeared ($F(1,4) = 6.3,\ P < 0.05$, one tailed) with left targets slower than right targets, and a significant interaction of task and side of target ($F(1,4) = 9.6,\ P < 0.05$, with more neglect being seen on the reach task.

### 2.6. *Results – interleaved tasks*

The median RTs to correctly perform these tasks are shown in Figs 4(a) and (b). Patients BK, and GL performed without errors, and NS made a single error on the say task. In contrast SJ and TH made high levels of errors and so their error rates to the different conditions

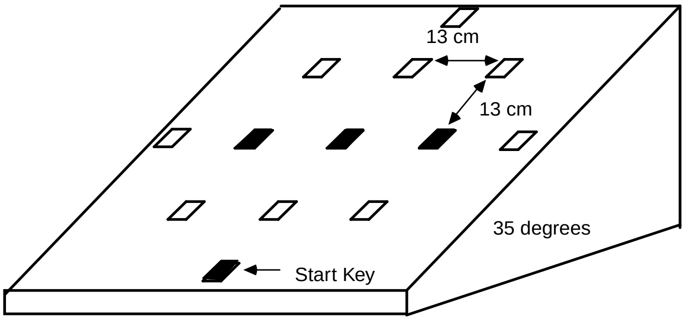

Fig. 2. The apparatus used for this study. The four keys used here are shown in black.

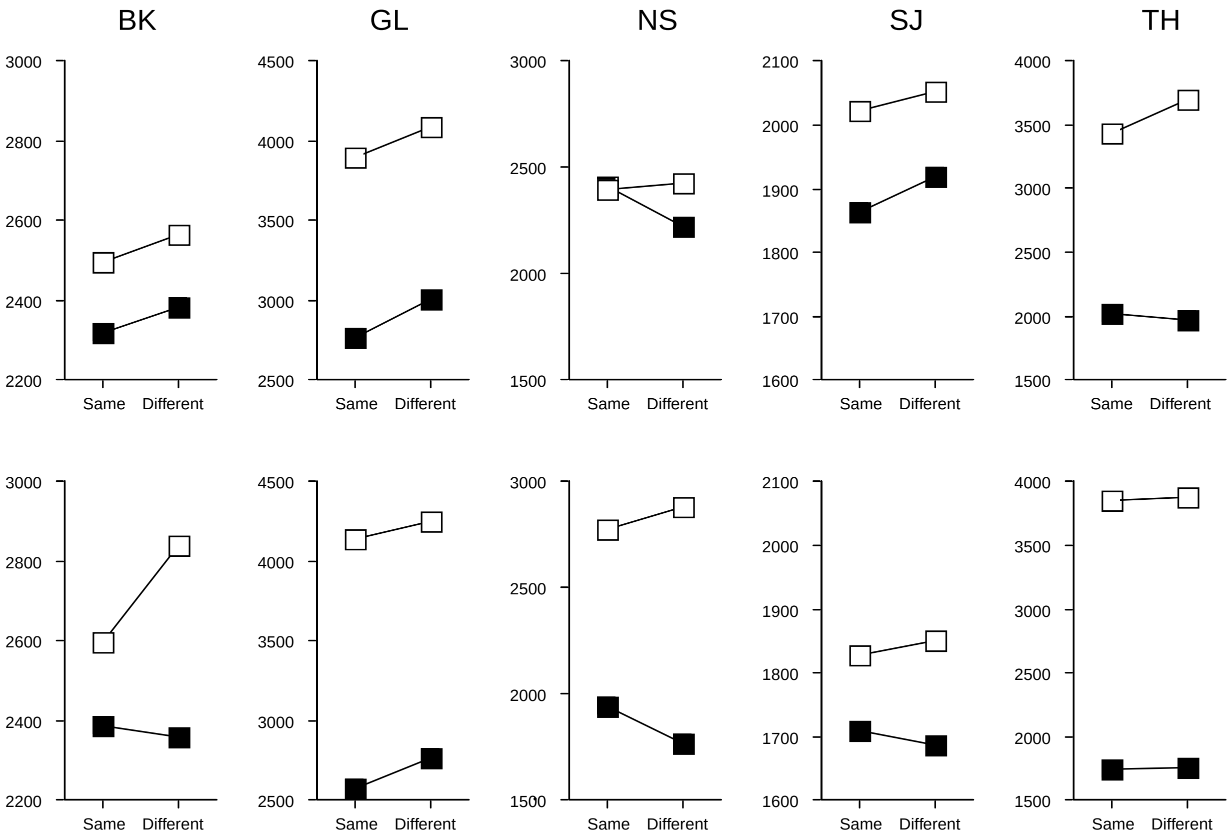

Fig. 3. The median RTs to perform the say task (above) and reach task (below) in each of the patients. Patients made no errors on these tasks. Data for stimuli on the left are shown as open symbols, those for stimuli on the right are shown with filled symbols.

are also shown in Fig. 4(b). The individual reaction times on correct trials for each patient were subjected to a within-subject analysis of variance (ANOVA) with the four factors side, side-change, task and task-change. The results of these five ANOVAs are summarized in Table 1, which shows the significance level of the different factors in each patient.

The results of these analyses are clear. In every case there was an effect of side, with response to the left having much longer RTs than responses to the right. In other words, all patients showed clear neglect. The other factor that had a significant effect on all patients was that of task-change. In other words, for all patients, for both tasks, the RT to make a reach or verbal response was longer when a different response was made on the previous trial. More important, the interaction of this factor with the factor of side was significant in all cases. The source of this interaction is clear by inspection of

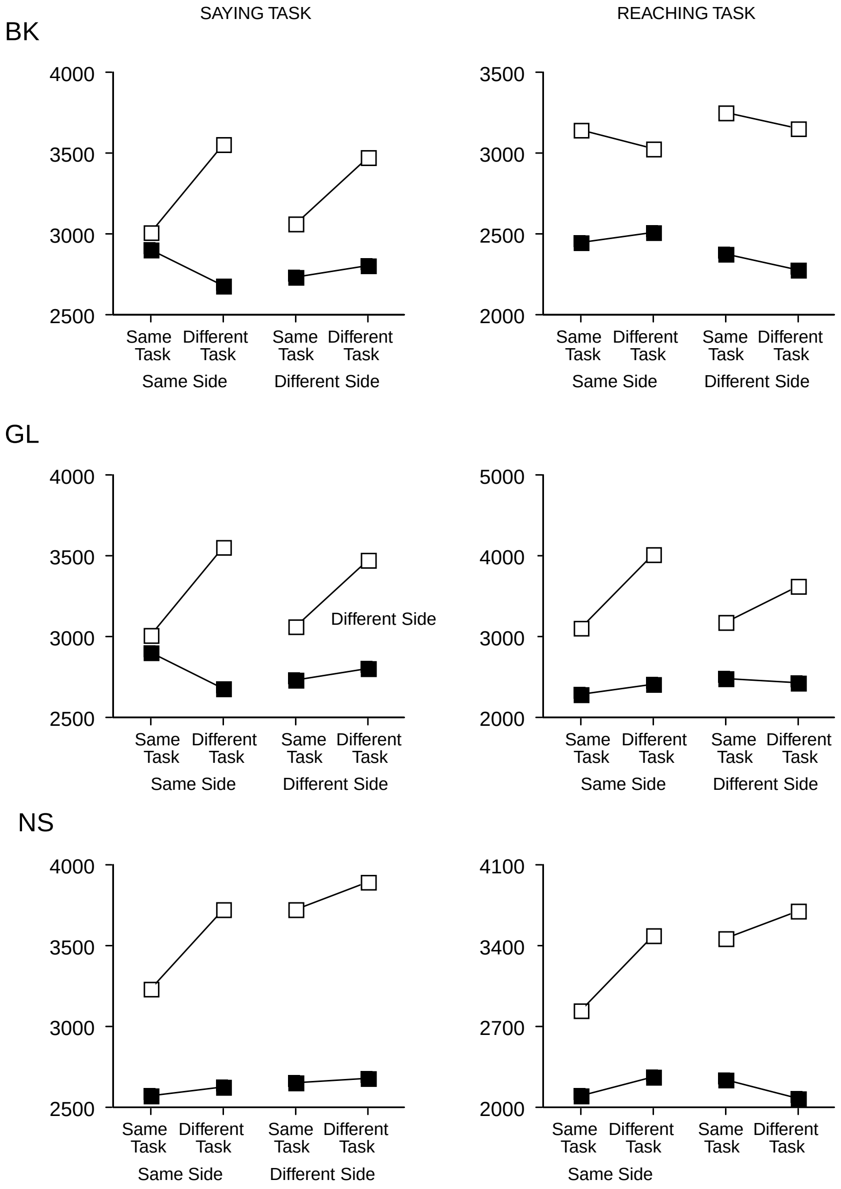

Fig. 4. The mean RTs to perform the mixed reach and say task. (a) for patients BK, GL and NS; patients BK and GL did not make any errors on this task. NS made a single error on the say task. (b) the mean RTs and error rates of SJ and TH on the mixed reach and say task. The data for the individual components (reaching and saying) are shown separately, with data from the say task on the left in each case, and data from the reach task on the right. Data for stimuli on the left are shown as open symbols, those for stimuli on the right are shown with filled symbols.

Figs 4(a) and (b) – there is only an effect of task-change for targets in the left visual field. No other factors were significant in all patients, although three patients showed an effect of task (BK and GL were faster on the reach task, while SJ was faster on the say task).

In order to test whether task-change affected only responses in the left side of the apparatus we carried out separate analyses for the left and right targets, analyzing simply for the factor of task-change. In every case, there was a significant effect of task change for items on the left of the display, but not for items on the right.

## 3. Discussion

Patients with lesions to the right parietal lobe leading to left visual neglect were tested on two tasks requiring detection of targets in the left and right of visual space.

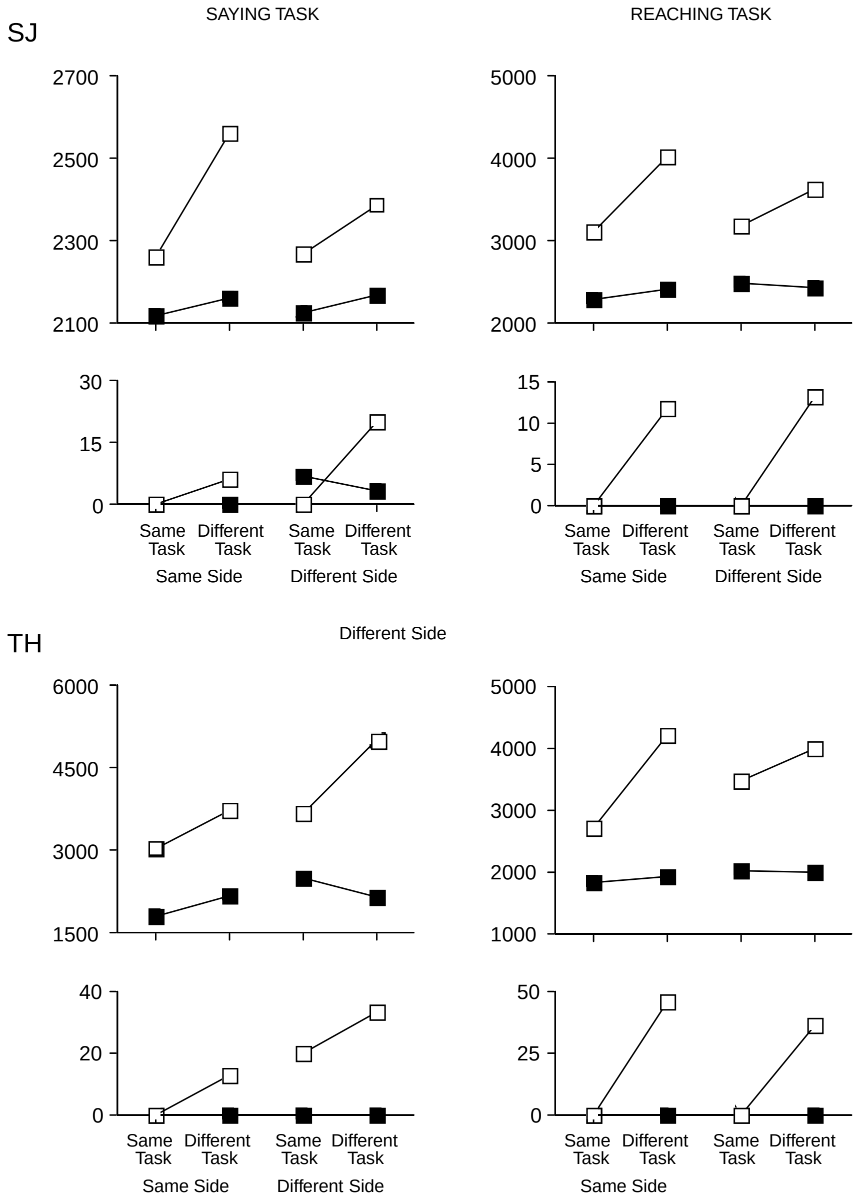

Fig. 4. continued.

In the reach task, patients had to detect and reach to touch an illuminated key that could be in the left or right of fixation. In the say task, targets appeared in the exact same spatial locations, and yet patients had to make a verbal response as to their location – saying "left" or "right" as quickly as possible. These two tasks were performed with ease by all patients, although reaction times to targets in the left hemifield were longer, due to a relative inattention to the left side of space.

When the two tasks of reaching and verbal response to stimuli were randomly interleaved, patients showed increased reaction times to make responses to a left target when the previous response had been different. Thus, for example, reaching responses to the left that followed a verbal response to a left stimulus led to longer reaction times than a reaching response that immediately followed a reaching response to the left. This cost of changing the action only occurred on the left side of the display, suggesting that it was a deficit that resulted from the lesions to the right parietal lobe. These results suggest that the parietal cortex is required not just to code a location in space [1], but also has a

Table 1
The results of the individual ANOVAs performed on the data for each of the five patients. Factors significant at a level less than 0.001 are indicated by ***, 0.01 is represented by **, 0.05 is represented by *. Trends at approximately 0.06 are indicated by (*), non-significant cases are left blank

| | BK | GL | NS | SJ | TH |
|---|---|---|---|---|---|
| side | *** | *** | *** | *** | *** |
| task-change | * | ** | * | * | *** |
| side x task-change | * | * | (*) | ** | *** |
| task | *** | *** | | * | |
| task x side | | (*) | | (*) | |
| task x task-change | * | | | | |
| side x side-change | | * | | | |
| side-change x task-change | | | | * | |
| task x side x side-change | | * | | | |
| task x side x task-change | * | | | | |

role in coding the action that must be performed to an object at that location in space.[1]

This can also be seen as support for views that attention is action-based (e.g. [31,33]). That is to say, the notion of attention not only applies to the location or object that is to be attended, but also crucially includes the action that is to be performed to that object. Our results suggest that the action-based nature of attention may be largely the result of the coding of both action and location by the parietal lobe. This result can be understood in a straightforward manner, given the results of Andersen and colleagues [12,24,28,29]. The posterior parietal cortex of the macaque contains neurons that code the location in space of a target that needs to be reached for, or else looked at. As such, these neurons could be described as forming the neural substrate of attention to those locations. However, the neurons of this region of the primate brain also code the action that must be carried out (reaching or saccades). Thus, the parietal cortex can be seen as also coding spatial intention.

Given these findings in the macaque brain, the effects of lesions to this region of the human brain may be understood. In the lesioned hemisphere, responses to contralesional targets will be produced slowly, since a diminished neuronal population is available. Since a different action requires the recruitment of an entirely different (lesioned) neuronal population, this might be expected to lead to longest reaction times when a new action has to be encoded. It might be argued that the effect of task change simply represents an exaggeration of the left-right asymmetry on those trials when response was more difficult, or more resource-demanding. Such an effect might be described in terms of central load effects on inattention, rather than as a deficit in intention (see, e.g. [23]). However, it should be noted that these trials are only difficult precisely because the action to be performed at a particular location is different. Our findings are distinct from work on showing an involvement of the parietal lobe in task-switching (see, for example [**?**,20]). In a task switching paradigm, subjects typically have to change between different stimulus to response mappings, whereas in the present study, S-R mappings are preserved throughout. On trials when a patient has particular difficulty, it is simply the switch to a new motor act. We suggest that it is the requirement to generate a new motor act that is the source of the deficit.

In summary, we have shown that patients with lesions to the parietal cortex show the greatest deficit when they are required to carry out an action that is different to that previously performed. This deficit in intention applies only to the contralesional side of space. These results show that the parietal cortex of the human is necessary not just for paying attention to particular spatial locations, but also codes the action that will be performed to a particular target in space.

## Author notes

This work was supported by a grant from the National Science Foundation (SBR 96-16555) and by generous intramural funding from the University of South Carolina. We thank all the patients who generously volunteered their time in this study. Correspondence concerning this article may be addressed to Gordon C. Baylis at: Attention and Perception Laboratory, Department of Psychology, University of South Carolina, Columbia, SC 29208. Email to gordon@sc.edu.

## References

[1] R.A. Andersen, Multimodal integration for the representation of space in the posterior parietal cortex, *Phil Trans Roy Soc* **B 352** (1999), 1421–1428.
[2] F. Attneave, Triangles as ambiguous figures, *American Journal of Psychology* **81** (1968), 447–453.
[3] G.C. Baylis and L.L. Baylis, Visual misguided reaching in Balint's Syndrome, *Neuropsychologia* **39** (2001), 865–875.

[1] Note that some views of spatial action might suggest that a verbal response does not qualify as a spatial action. However, it remains true that this is an action that must be programmed in response to an item at a particular spatial location. Thus it should not come as a surprise that such an action is subject to the same constraints of intention as a spatial reaching action.

[4] G.C. Baylis and L.L. Baylis, Effects of brain damage on selective reaching, in: *Selection and Action*, S. Jackson, ed., University College London Press, 2002.

[5] G. Baylis, J. Driver and R. Rafal, Visual extinction and stimulus repetition, *Journal of Cognitive Neuroscience* **5**(4) (1993), 453–466.

[6] G.C. Baylis and C.J. Gore, Task set can determine the reference frame for neglect, *Neuropsychologia* (2002).

[7] G.C. Baylis, C.L. Gore, P.D. Rodriguez and R.J. Shisler, Visual extinction: the importance of binding dorsal and ventral visual pathways, *Visual Cognition* **B** (2001), XX–XX.

[8] M. Behrmann and S.P. Tipper, Attention accesses multiple reference frames: evidence from visual neglect, *Journal of Experimental Psychology: Human Perception & Performance* **25**(8) (1999), 359–379.

[9] E. Bisiach and G. Vallar, Hemineglect in humans, in: *Handbook of Neuropsychology,* (Vol. 1), F. Boller and J. Grafman, eds, Elsevier, Amsterdam, 1988, pp. 195–222.

[10] E. Bisiach, G. Geminiani, A. Berti and M.L. Rusconi, Perceptual and premotor factors of unilateral neglect, *Neurology* **40** (1990), 1278–1281.

[11] E. Bisiach, R. Ricci, M. Lualdi and M.R. Colombo, Perceptual and response bias in unilateral neglect, *Brain and Cognition* **37** (1998), 369–386.

[12] R.M. Bracewell, P. Mazzoni, S. Barash and R.A. Andersen, Motor intention activity in the macaque's lateral intraparietal area. I. Changes of motor plan, *J. Neurophysiol.* **76** (1996), 1457–1464.

[13] J. Driver, G.C. Baylis, S. Goodrich and R. Rafal, Axis-based neglect of visual shapes, *Neuropsychologia* **32** (1994), 1353–1365.

[14] J. Driver, G.C. Baylis and R. Rafal, Preserved figure-ground segregation and symmetry perception in visual neglect, *Nature* **360** (1992), 73–75.

[15] M.J. Farah, J.L. Brunn, A.B. Wong, M.A. Wallace and P. Carpenter, Frames of reference for allocating attention to space: Evidence from the neglect syndrome, *Neuropsychologia* **28** (1990), 335–347.

[16] M. Husain, J.B. Mattingley, C. Rorden, C. Kennard and J. Driver, Distinguishing sensory and motor biases in parietal and frontal neglect, *Brain* **123** (2000), 1643–1659.

[17] S.R. Jackson, R. Newport, M. Husein, M. Harvey and J. Hindle, Reaching movements may reveal the distorted totgraphy of spatial representations after neglect, *Neuropsychologia* **38** (2000), 500–507.

[18] P.B. Johnson, S. Ferraina and R. Caminiti, Cortical networks for visual reaching, *Exp Brain Res* **97**(2) (1993), 361–365.

[19] B.M. De Jong, A.T. Willemsen and A.M. Paans, Brain activation related to the change between bimanual motor programs, *Neuroimage* **9** (1999), 290–297.

[20] D.Y. Kimberg, G.K. Aguirre and M. D'Esposito, Modulation of task-related neural activity in task-switching: and fMRI study, *Brain Res: Cogn brain Res* **10** (2000), 189–196.

[21] M. Matelli, R. Camarda, M. Glickstein and G. Rizzolatti, Afferent and efferent projections of the inferior area 6 in the macaque monkey, *J Comp Neurol* **251**(3) (1986), 281–298.

[22] J.B. Mattingley, M. Husain, C. Rorden, C. Kennard and J. Driver, Motor role of human inferiror parietal lobe revealed in unilateral neglect patients, *Nature* **392** (1998), 179–182.

[23] E.A. Maylor and N. LaVie, The effect of perceptual load on age differences in selective attention, *Psychology and Aging* **13** (1998), 563–573.

[24] P. Mazzoni, R.M. Bracewell, S. Barash and R.A. Andersen, Motor intention activity in the macaque's lateral intraparietal area. I. Dissociation of motor plan from sensory memory, *J. Neurophysiol.* **76** (1996), 1439–1456.

[25] V.B. Mountcastle, J.C. Lynch, A. Georgopoulos, H. Sakata and C. Acuna, Posterior parietal association cortex of the monkey: command functions for operations within extrapersonal space, *J Neurophysiol* **38**(4) (1975), 871–908.

[26] C. Rorden and Brett, MRIcro software: Stereotaxic display of brain lesions, *Behavioral Neurology* (2001), (in press).

[27] P. Simone and G.C. Baylis, Effects of Alzheimer's Disease on Attention, *Journal of Experimental Psychology: Human Perception and Performance* **23** (1997), 595–608.

[28] L.H. Snyder, A.P. Batista and R.A. Andersen, Coding of intention in posterior parietal cortex, *Nature* **386** (1997), 167–170.

[29] L.H. Snyder, A.P. Batista and R.A. Andersen, Intention-related activity in the posterior parietal cortex: a review, *Vision Research* **40** (2000), 1433–1441.

[30] M.H. Sohn, S. Ursu, J.R. Anderson, V.A. Stenger and C.S. Carter, Inaugural article: The role of prefrontal cortex and posterior parietal cortex in task switching, *Proc Natl. Acad. Sci.* **97** (2000), 13448–13453.

[31] S.P. Tipper, L.A. Howard and G. Houghton, Action-based mechanisms of attention, *Philos Trans R Soc Lond* **B353** (1998), 1385–1393.

[32] S.P. Tipper and M. Behrmann, Object-centered not scene-based visual neglect, *Journal of Experimental Psychology: Human Perception & Performance* **22**(5), 1261–1278.

[33] S.P. Tipper, C. Lortie and G.C. Baylis, Selective reaching: Evidence for action-centered attention, *Journal of Experimental Psychology: Human Perception and Performance* **18** (1992), 891–905.

[34] D. Zipser and R.A. Andersen, A back-propagation programmed network that simulates response properties of a subset of posterior parietal neurons, *Nature* **331**(6158) (1988), 679–684.
